# Early mobilisation after transfemoral transcatheter aortic valve implantation: results of the MobiTAVI trial

**DOI:** 10.1007/s12471-020-01374-5

**Published:** 2020-02-28

**Authors:** J. Vendrik, W. Vlastra, M. S. van Mourik, R. Delewi, M. A. Beijk, J. Lemkes, J. J. Wykrzykowska, R. J. de Winter, J. S. Henriques, J. J. Piek, M. M. Vis, K. T. Koch, J. Baan Jr.

**Affiliations:** 1 Heart Centre, Amsterdam University Medical Centres (location AMC), Amsterdam, The Netherlands; 2 Heart Centre, Amsterdam University Medical Centres (location VUMC), Amsterdam, The Netherlands

**Keywords:** Transfemoral transcatheter aortic valve implantation, Percutaneous valve, Safety protocol, Early mobilisation, Ambulation, Vascular complications

## Abstract

**Background:**

Immobilisation of patients after transfemoral transcatheter aortic valve implantation (TF-TAVI) is the standard of care, mostly to prevent vascular complications. However, immobilisation may increase post-operative complications such as delirium and infections. In this trial, we determine whether it is feasible and safe to implement early ambulation after TF-TAVI.

**Methods:**

We prospectively included TF-TAVI patients from 2016 to 2018. Patients were assessed for eligibility using our strict safety protocol and were allocated (based on the time at which the procedure ended) to the EARLY or REGULAR group.

**Results:**

A total of 150 patients (49%) were deemed eligible for early mobilisation, of which 73 were allocated to the EARLY group and 77 to the REGULAR group. The overall population had a mean age of 80 years, 48% were male with a Society of Thoracic Surgeons Predicted Risk of Mortality (STS-PROM) score of 3.8 ± 1.8. Time to mobilisation was 4 h 49 min ± 31 min in the EARLY group versus 20 h 7 min ± 3 h 6 min in the REGULAR group (*p* < 0.0001). There were no differences regarding the primary endpoint. No major vascular complications occurred and a similar incidence of minor vascular complications was seen in both groups (4/73 [5.5%] vs 6/77 [7.8%], *p* = 0.570). The incidence of the combined secondary endpoint was lower in the EARLY group (*p* = 0.034), with a numerically lower incidence for all individual outcomes (delirium, infections, pain and unplanned urinary catheter use).

**Conclusion:**

Early mobilisation (ambulation 4–6 h post-procedure) of TF-TAVI patients is feasible and safe. Early ambulation decreases the combined incidence of delirium, infections, pain and unplanned urinary catheter use, and its adoption into contemporary TAVI practice may therefore be beneficial.

**Electronic supplementary material:**

The online version of this article (10.1007/s12471-020-01374-5) contains supplementary material, which is available to authorized users.

## What’s new?

- Early mobilisation (ambulation 4–6 h post-procedure) is feasible after contemporary lower-risk transfemoral transcatheter aortic valve implantation (TF-TAVI).
- Early ambulation, after strictly selecting eligible TF-TAVI patients, was associated with a similar rate of vascular complications when compared to the standard protocol (supine bed rest until the next morning).
- Early ambulation after TF-TAVI lowers the combined incidence of delirium, infections, pain and unplanned urinary catheter use.
- It may be beneficial to adopt early mobilisation into contemporary TF-TAVI practice.

## Introduction

Transcatheter aortic valve implantation (TAVI) is the preferred treatment for severe symptomatic aortic valve stenosis in inoperable and high-risk patients, and has been proven to be a non-inferior alternative for surgical valve replacement (SAVR) in intermediate-risk patients [1–4]. Transfemoral (TF)-TAVI may be superior to SAVR in the latter population [5]. The gradual broadening of indications for TF-TAVI now extends to even low-surgical-risk patients, accordingly to the results of the Low-Risk TAVR (LRT) trial and the recently published results from the ‘Placement of Aortic Transcatheter Valves (PARTNER) III’ and ‘Medtronic Evolut Transcatheter Aortic Valve Replacement in Low Risk Patients’ trials comparing TAVI and SAVR in low-risk patients [6–8].

Secondary outcomes such as physical and cognitive functioning, quality of life in the remainder of the patient’s life and in-hospital comfort are becoming of greater importance and interest in younger and healthier patients. However, vascular and bleeding complications can severely impair these outcomes. Post-procedural immobilisation is the standard of care to prevent these complications after TF-TAVI. However, unnecessarily long immobilisation may increase the incidence of other post-operative complications such as delirium and infections, and may cause patient discomfort and raise healthcare costs. Post-operative delirium and infection are both associated with a significantly worsened clinical outcome after TAVI [9–12]. Since the transfemoral route allows the practice of ‘minimalist’ TAVI, i.e. a fully percutaneous access by applying local or conscious sedation, it allows rapid recovery and a short hospital stay [13–17].

Early mobilisation may lower the incidence of post-operative delirium, infection and patient’ discomfort. However, contemporary practice varies widely regarding both immobilisation and hospitalisation after TF-TAVI [18]. Previous studies on early ambulation after transfemoral cardiac interventions such as coronary angiography and percutaneous coronary intervention showed no increase in vascular complications (haematoma and access site bleeding) when comparing early versus late or standard ambulation [19–21]. These studies obviously concerned a different population and much smaller sheath sizes used for access.

In this trial, we assessed the safety and feasibility of an early ambulation protocol after TF-TAVI. Moreover, we evaluated potential patient benefits of early ambulation on the incidence of in-hospital complications such as delirium, infections, pain, unplanned urinary catheter use and, lastly, the duration of the hospital stay.

## Methods

### Inclusion criteria

We prospectively included all consecutive patients undergoing TF-TAVI from September 2016 until August 2018 at the Amsterdam University Medical Centre (Amsterdam UMC, location AMC), a high-volume tertiary centre in Amsterdam, the Netherlands. In patients with symptomatic aortic valve stenosis, decisions regarding treatment, access route and valve selection were at the discretion of our multidisciplinary TAVI team. These decisions were part of regular clinical care and based on pre-operative screening, including computed tomography angiography, cardiac echocardiography and diagnostic coronary catheterisation, all performed in accordance with the most recent guidelines [19, 20]. After the decision to perform TAVI using the transfemoral approach, patients were assigned randomly to two pre-defined weekdays at the discretion of our planning bureau, which had no insight into the expected difficulty of the procedure or the patients’ health status. The operators were assigned to the two pre-defined week days weeks before the patients were. The Institutional Review Board approved this study with a waiver, and the trial was registered in the Dutch Trial Register (NTR 6098).

### Procedure and vascular closure

The standard approach for TAVI was a fully percutaneous transfemoral approach using local anaesthesia. We followed regular hospital protocol regarding the pre-procedural administration of heparin and protamine, based on the weight of the patients and the measured activated clotting time. For vascular closure, the double-ProGlide preclose technique (Abbott Vascular, CA, USA) and the Manta closure device (Essential Medical, Exton, PA, USA) were used for valve introduction [21–24]. The non-valve side was closed with either a single ProGlide or an Angio-Seal (Terumo Medical Corporation, NJ, USA). Afterwards, SafeGuards (Merit Medical, South Jordan, UT, USA) were placed on both groins; the devices were deflated after 2 h and removed after 4 h according to hospital protocol.

### Patient eligibility and treatment allocation

**Fig. 1:**
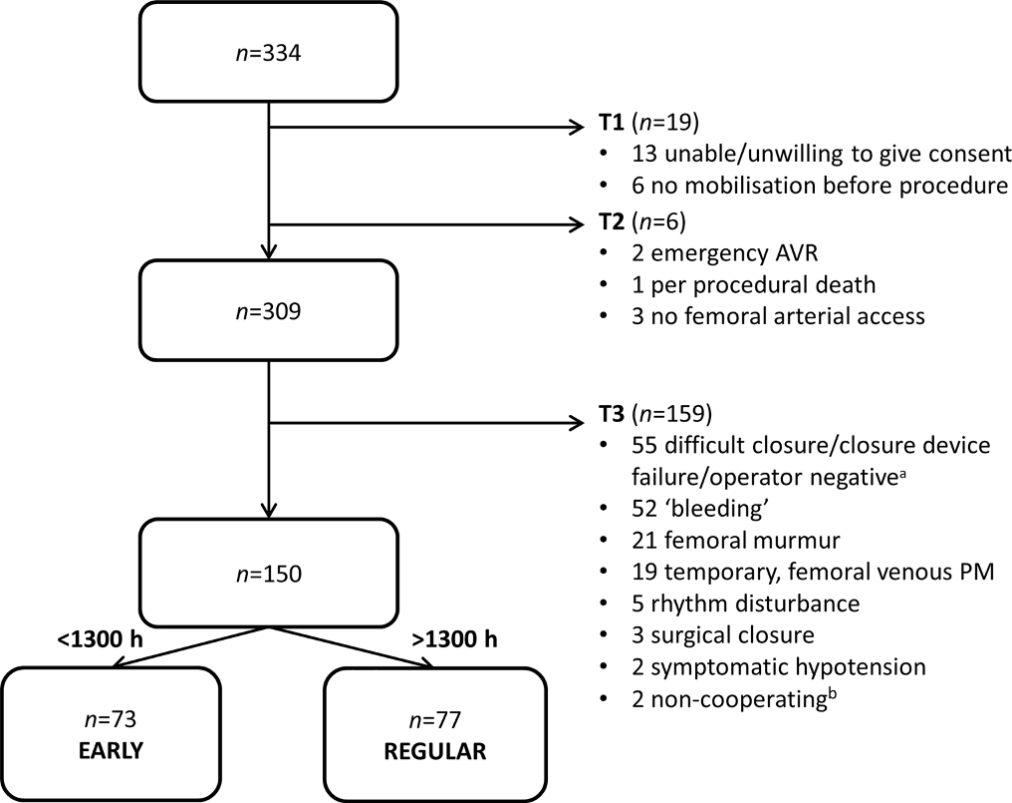
Flowchart of study patient selection. (*T1* pre-TAVI, *T2* during procedure, *T3* 4 h after the procedure, *AVR* aortic valve replacement, *PM* pacemaker, *TAVI* transcatheter aortic valve implantation. ^a^Operator recommended not including the patient in the early ambulation group. ^b^Two eligible patients were not willing to ambulate early)

We developed a strict protocol to assess patient eligibility for early mobilisation and to guarantee patient safety. Patients could be excluded at three different time points during the hospital stay (Fig. 1; see Electronic Supplementary Material, Table S1, for complete checklist). The first time point, T1, was assessed before the procedure, whereas T2 was assessed during and directly after the procedure. After 4 h, following consultation with the operator and physical examination of the patient, T3 was assessed. After passing the three time points, the patient was deemed eligible for early mobilisation and was either allocated to the early mobilisation group (EARLY), i.e. ambulation within 4–6 h after the procedure, or to the regular hospital protocol (REGULAR), which consisted of supine bed rest until the next morning. Allocation was performed based on the time at which the procedure ended; all patients in whom the procedure was finished before 1,300 hours were allocated to the EARLY group, and all patients after 1,300 hours to the REGULAR group. The reason for choosing this design was twofold: (1) to increase clarity and feasibility for the medical staff and (2) to increase the safety of the patients in the EARLY group, who in this manner would ambulate during the fully staffed day shift.

### Outcomes

Baseline characteristics including data from the pre-operative screening were prospectively collected in the AMC TAVI database. The primary endpoint of this trial was the safety of early ambulation, consisting of the presence of vascular (access site) complications and access site bleedings (according to the VARC‑2 criteria [22]). The secondary endpoint was the combined incidence of in-hospital outcomes. In-hospital outcomes included post-operative pain, scored with the Visual Analogue Scale (VAS, whereby post-operative pain was defined as VAS >3 [23, 24]), post-operative delirium (confirmed by a geriatric internist), clinically diagnosed infections (defined as the clinical suspicion with conclusive laboratory [increase in C‑reactive protein or leucocytes] or conclusive microbiology findings), and unplanned urinary catheter use (defined as urinary catheter use in patients who were hospitalised without a urinary catheter before TAVI). As a secondary safety endpoint, fall incidents were registered. Lastly, the duration of the hospital stay was evaluated and was defined as the number of days from the TF-TAVI to the day the patient was discharged to home.

### Statistical analysis

The primary and the secondary endpoint were compared between the EARLY and REGULAR group. The secondary endpoint was analysed as a composite of the in-hospital complications (incidence of pain, infection, delirium and unplanned urinary catheter use). Moreover, all individual in-hospital outcomes were compared between the EARLY and REGULAR group. Categorical variables are presented as numbers with percentages and compared between both groups using Pearson’s chi-squared test. Continuous data were checked for normality, and are presented as mean with standard deviation or median with interquartile range and compared using an unpaired Student’s *t*-test or Mann-Whitney U‑test as appropriate. A double-sided *p*-value <0.05 was considered significant. All analyses were performed using SPSS software (version 24.0 for Windows, SPSS, Inc., Chicago, IL, USA). Since this was a first-time study and no comparable studies are available, no reasonable assumptions could be made regarding the expected incidences of the primary and secondary outcomes. Therefore, we did not perform a sample size analysis for the primary or the secondary endpoint.

## Results

### Study population and patient eligibility

The flowchart of patient distribution at the different time points and allocation to subgroup are shown in Fig. 1. The total study population consisted of 309 patients who underwent successful TF-TAVI, ambulating before the procedure and consenting to study participation. The main reason for ineligibility and thus exclusion 4 h after the procedure (T3) was that possible early ambulation was considered to be too hazardous, because of difficult vascular closure (*n* = 55, 35%), as decided by the operator. Nine of 159 (5.7%) of these patients had a closure device failure according to the VARC-II criteria. Thereafter, residual bleeding/‘oozing’ (*n* = 53, 33%), the presence of any systolic femoral murmur (*n* = 21, 13%) and the presence of a transvenous temporary pacemaker (*n* = 19, 12%) were the most prominent reasons for exclusion after 4 h. In the 21 patients deemed ineligible because of a systolic femoral murmur, a false aneurysm was found in 7 patients and was treated accordingly.

The eligible population had a mean age of 80 years, 48% were male and had a mean Society of Thoracic Surgeons Predicted Risk of Mortality (STS-PROM) score of 3.781 ± 1.842, reflecting contemporary practice in a lower-risk TF-TAVI population. These 150 eligible patients were allocated to either the EARLY (*n* = 73) or REGULAR group (*n* = 77), as previously described. Two eligible patients were not willing to ambulate early; no further ‘cross-over’ happened between the EARLY and REGULAR group. Baseline characteristics of the subgroups are shown in Tab. 1, and were equally distributed in the subgroups, except for a lower EuroSCORE II and slightly better estimated renal function (expressed as estimated glomerular filtration rate) in the EARLY group. There were no significant differences in pre-procedural medical regimen (i.e. anti-aggregation or anti-coagulation) between the two subgroups.

**Table 1:** Baseline characteristics of EARLY versus REGULAR group

	EARLY (*n* = 73)	REGULAR (*n* = 77)	*p*-value
Age	78.92 ± 10.9	80.47 ± 6.2	0.624
Men	40 (55%)	32 (42%)	0.105
BMI	27.1 ± 4.8	28.3 ± 6.0	0.183
AVA	0.78 ± 0.18	0.80 ± 0.19	0.567
Peak AV gradient	65 ± 25	62 ± 20	0.404
STS-PROM	3.522 ± 1.845	4.028 ± 1.818	0.092
EuroSCORE II	2.72 ± 1.55	3.71 ± 2.14	0.001
DM	23 (32%)	27 (35%)	0.644
COPD	8 (11%)	12 (16%)	0.405
AF	27 (37%)	22 (29%)	0.272
Previous CABG	3 (4%)	8 (10%)	0.147
Previous PCI	13 (18%)	22 (29%)	0.130
Previous stroke	6 (8%)	8 (10%)	0.667
Previous PM	8 (11%)	4 (5%)	0.193
Creatinine (µmol/l)	94 ± 43	108 ± 60	0.124
eGFR	61 ± 17	54 ± 16	0.011

All data presented as mean ± standard deviation or as number of patients and percentage of subgroup

*AF* atrial fibrillation, *AV gradient* aortic valve gradient (mm Hg), *AVA* aortic valve area (cm^2^), *BMI* body mass index (kg/m^2^), *CABG* coronary artery bypass grafting, *COPD* chronic obstructive pulmonary disease, *DM* diabetes mellitus, *PCI* percutaneous coronary intervention, *PM* pacemaker, *eGFR* glomerular filtration rate (using the MDRD formula, presented as ml/min/1.73 m^2^), *STS-PROM* Society of Thoracic Surgery—predicted risk of mortality

### Procedural characteristics

Procedural characteristics and outcome are shown in Tab. 2 and were similarly distributed in the EARLY and REGULAR group. The vast majority of the patients were treated using the third-generation balloon-expandable SAPIEN 3 (Edwards Lifesciences, Irvine, CA, USA) prosthesis (95%), with similar distribution in the two groups regarding valve type and valve size. All patients were treated using a fully percutaneous approach and local analgesia only. For arterial closure on the valve introduction side, double ProGlides were most frequently used. For the contralateral side, a single ProGlide or an Angio-Seal was used most frequently.

**Table 2:** Procedural characteristics, primary and secondary endpoints for EARLY and REGULAR group

	EARLY (*n* = 73)	REGULAR (*n* = 77)	*p*-value
SAPIEN 3	68 (93.2%)	74 (96.1%)	0.309
Valve size distribution (20/23/26/29 mm)^a^	0/24/25/13	2/32/29/10	0.367
Arterial closure valve side (double Proglide/single Proglide/Manta/Prostar)	62/1/5/5	66/2/2/7	0.550
Arterial closure non-valve side (single Proglide/Angioseal/none)	30/41/2	35/40/2	0.865
Time to mobilisation	4 h 49 min ± 31 min	20 h 7 min ± 3 h 6 min	<0.0001
*Primary endpoint:*			
Major vascular complications	0	0	–
Major bleeding complications	0	0	–
Minor vascular complications	4 (5.5%)	6 (7.8%)	0.570
Minor bleeding	4 (5.5%)	6 (7.8%)	0.570
*Secondary endpoints:*			
Pain^b^	4 (5.5%)	8 (10.4%)	0.218
Infection	2 (2.7%)	3 (3.9%)	0.693
Delirium	1 (1.4%)	2 (2.6%)	0.591
Unplanned urinary catheter use	3 (4.1%)	8 (10.4%)	0.140
– Combined endpoint^c^	9 (12.3%)	20 (26.0%)	0.034
Prolonged hospitalisation^d^	30 (41.1%)	40 (51.9%)	0.183
Duration of hospital stay (median days [IQR])	3 (2–5)	4 (2–6)	0.243

All data are presented as mean ± standard deviation or as number of patients and percentage of subgroup

^a^Only for the SAPIEN 3

^b^Presence of pain the next morning is defined as a Numerical Rating Scale/Visual Analogue Scale score >3 during the start of the day shift

^c^Combined secondary endpoint: incidence of pain, infection, delirium and urinary catheter use (some patients had >1 endpoint)

^d^Defined as post-procedural hospital stay >3 days

### Outcome

**Fig. 2:**
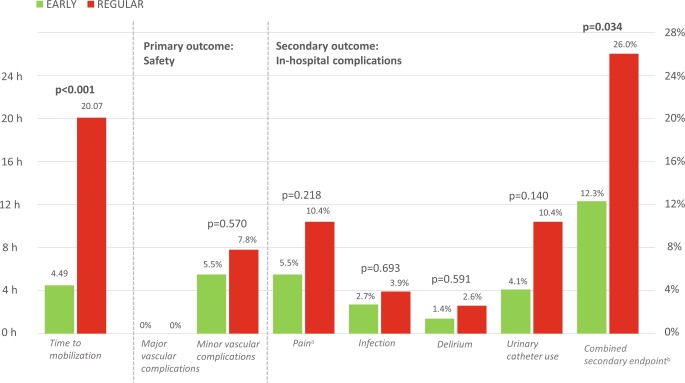
Primary and secondary endpoint: in-hospital outcomes for EARLY versus REGULAR group. (^a^Presence of pain the next morning is defined as a Numerical Rating Scale/Visual Analogue Scale score >3 during the start of the day shift. ^b^Combined secondary endpoint: incidence of pain, infection, delirium and unplanned urinary catheter use (some patients had >1 endpoint))

The outcomes regarding the primary and secondary endpoint are shown in Fig. 2 and Tab. 2. Time to mobilisation was four-fold longer in the patients following regular hospital protocol (4 h 49 min ± 31 min vs 20 h 7 min ± 3 h 6 min for EARLY vs REGULAR, *p* < 0.0001). There was no difference regarding the primary (safety) endpoint between the EARLY and REGULAR group. No major vascular or bleeding complications occurred in either group. The incidence of minor vascular complications, all minor bleedings, was similar in both groups (5.5% vs 7.8% for EARLY vs REGULAR, respectively, *p* = 0.570).

The overall incidence of severe pain the next morning (8.0%), infection (3.3%), delirium (2.0%) and the need for a urinary catheter (7.3%) was low. No fall incidents occurred. Regarding the secondary endpoint, a significantly lower combined incidence of the in-hospital outcomes was seen, favouring the EARLY group (12.3% vs 26.0%, *p* = 0.034). All individual in-hospital outcomes were numerically lower in the EARLY group.

Lastly, the duration of the hospital stay in the total study cohort was relatively short (median 3 days) and statistically similar in the EARLY and REGULAR group.

## Discussion

In the current trial, early ambulation protocol following TF-TAVI after strict selection of patients using our safety protocol was associated with a comparable rate of vascular complications. This indicates that such a selection and early ambulation protocol is feasible and safe to perform after contemporary TF-TAVI.

### Study population and patient eligibility

Of the total cohort, 49% were deemed eligible for early mobilisation. Since this was a first-time trial, we predominantly focused on feasibility and safety, and thus were very strict in excluding patients considered to be at increased risk for complications after possible early ambulation. This cautious approach was also taken for the actual ambulation, which was performed under direct supervision of the nursing staff, taking into consideration the increased risk for falling incidents in this elderly, frail population. Considering the low number of minor vascular complications and the total absence of fall incidents, we succeeded in selecting patients for safe early ambulation. We believe that these results could be extrapolated to the patients who were treated in the afternoon, reasoned from the total absence of major complications (which require intervention by an interventional radiologist/vascular surgeon, preferably performed during daytime). Lastly, we report on a relatively low-risk population, when compared to large randomised trials like the PARTNER 2A and PARTNER 3, SURTAVI and CoreValve Low Risk Trial [3–7]. Accordingly, our results and protocol could be used in other hospitals to introduce the possibility of early mobilisation after contemporary lower-risk TF-TAVI.

Most of the excluded patients at T3 (*n* = 159, 4 h post-procedure) were deemed ineligible for early mobilisation because of a difficult arterial closure so that early mobilisation was considered hazardous. Of these patients, 9 of 159 (5.7%) had a closure device failure according to the VARC‑2 criteria [19]. These criteria state that a failed closure device placement only accounts for ‘closure device failure’ when another (second) closure device is used. The actual number of failed closure devices was higher (*n* = 32/159, 20.1% of the excluded patients; and 32/309, 10.3% of the total study population). These failing closure devices were treated with additional (manual) compression of the femoral artery, and patients were excluded accordingly, being at increased risk for bleeding complications in the case of early ambulation. Newer closure devices may increase the number of successful closures, especially in these old and calcified femoral arteries, and thus enlarge the proportion of patients eligible for early mobilisation [25–27]. Further studies could elaborate on the correlation between the quality of the peripheral vasculature (i.e. calcification burden) and the rate of successful closures, to ensure the maximum chance of successful closure and thus the possibility for early mobilisation.

The second reason for exclusion at T3 was residual ‘bleeding’, which was defined as any blood loss or active bleeding at the access site after 4 h (T3). Some of these cases probably were actually venous ‘oozing’, caused by the absence of a venous closure device. One could consider the possibility of adding a venous closure device to the procedural protocol, especially when used in combination with an additional cutaneous suture, which will increase eligibility for early mobilisation. Lastly, 21 patients were deemed ineligible because of a systolic femoral murmur; all underwent ultrasonography of the suspected femoral artery. In only 7 patients was a false aneurysm found and treated accordingly. In hindsight, the remaining 14 patients could have been eligible for early mobilisation, after the negative vascular ultrasound.

### Outcomes

In addition to the aforementioned ‘venous’ access site bleedings in the excluded patients, 2 of 4 and 2 of 6 vascular complications in the eligible patients allocated, respectively, to the EARLY and REGULAR group originated from the non-valve introduction side. These could have been related to either the secondary arterial access or to the venous access for the temporary pacemaker. Elimination of the contralateral access site by using radial arterial access and applying left-ventricular pacing via the stiff wire may increase eligibility for early mobilisation.

Our study indicates that early ambulation is safe, and shows a benefit of early mobilisation regarding the in-hospital secondary endpoint, showing a significant two-fold reduction in the incidence of the combined secondary outcomes. In particular, patients who ambulated early experienced less pain and less need for unplanned urinary catheter use, while being on supine bed rest for 15 h less than the patients following the regular protocol. We believe that this combination significantly improves patient comfort. Moreover, our study shows a trend in which early ambulation may potentially decrease the incidence of post-operative delirium and infections, hereby taking into consideration of the fact that we already show a very low incidence of these debilitating complications. These low incidences underline the effect of the practice of ‘minimalist TAVI’ using local analgesia only in contemporary TF-TAVI and, possibly, now subsequent ‘minimalist’ immobilisation.

The FAST-TAVI (NCT02404467) and 3M-TAVI (NCT02287662) provide us with the insights on how to reduce the length of hospital stay, and showing it can be done without any additional risks, supported by a recent systematic review by Kotronias et al. [14, 17, 18]. Our study adds to these results, since a patient needs to be able to ambulate properly in order to go home safely. In this way, our study forms the next step in improving and minimalising the TAVI procedure and subsequent hospitalisation. Our study does not show a reduction in the duration of the hospital stay when early ambulation is performed. This may be partially explained by the fact that the hospitalisation is a median of only 3 days after the procedure we describe, which is relatively short when compared to data in the current literature.

Lastly, while conducting this study we received some quite positive feedback from both patients and the medical staff. Although in-hospital comfort for staff and patients may not be easy to quantify, it is considered a valuable goal, especially when considering the growing number of procedures and patients’ expectations as well as requests for less invasive treatments. Therefore, early ambulation for eligible TF-TAVI patients was included in the regular hospital protocol at our centre directly after completion of the study.

### Future perspectives

We believe that ‘minimalist’ TAVI and subsequent ‘minimalist’ immobilisation and hospitalisation will be the standard form of care in the very near future, considering the broadening indication, accumulating evidence and exponential gain in experience worldwide [6, 7, 13, 18, 28]. Several procedural changes have already been introduced recently (i.e. local analgesia, fully percutaneous access) and even more could be introduced in the near future, further minimalising the contemporary TAVI procedure. Using left ventricular pacing (instead of transvenous right ventricular pacing) and the radial approach for the secondary arterial access (instead of the contralateral femoral artery) could further diminish vascular complications and increase eligibility for early mobilisation. Additionally, using the jugular vein for the temporary pacemaker lead could enable early mobilisation in patients who are pacemaker-dependent directly after the TF-TAVI. Lastly, technological advances in prostheses (and incrementally decreasing required sheath sizes) and closure devices may further enable early mobilisation in the majority of patients after TF-TAVI. Of these patients, the most elderly, fragile population will probably benefit the most from an early mobilisation protocol. However, the future lower-risk population would probably enlarge the proportion of eligible patients and accordingly increase the overall gain from an early mobilisation protocol. This gain, in combination with further simplifying the procedure and shortening the subsequent hospitalisation, will lower costs and will improve the cost-efficiency of contemporary TAVI.

### Limitations

First, this study was designed as a prospective trial with allocation of treatment based on the time of the procedure, and not as a truly randomised trial. We drafted this design predominantly for safety reasons, since this is the first time early ambulation has been studied in this elderly, frail TAVI population. In this manner we could ensure that the actual ambulation would be performed during the fully staffed day shift. The absence of randomisation could have introduced bias into the patient selection. However, patients were randomly allocated to two pre-defined weekdays by our planning bureau, who did not have any information about the expected complexity of the case or the health status of the patient. This led to a comparable patient population in the EARLY and REGULAR group.

Secondly, this is a single-centre study. This gave us the unique opportunity to perform this study safely. However, due to the relatively small sample size, this may have deprived us of the chance to find any significant differences proving a benefit of early mobilisation for the individual secondary outcomes. Larger, preferably multicentre studies are needed to demonstrate this patient benefit, showing favourable outcomes regarding debilitating post-operative complications like delirium and infections. Nevertheless, we do show a two-fold lower incidence of the combined secondary endpoint when early ambulation is used, which warrants the adoption of such a protocol into contemporary TAVI practice. Furthermore, we predominantly used ProGlides for vascular closure. Extrapolation of our study results should be performed with caution, especially when using different arterial closure methods or when there are different circumstances regarding nursing and medical staff during the day. The adjudication of events in this study was not blinded or performed by a Clinical Event Committee, which raises inherent limitations to our study.

## Conclusion

Early mobilisation (ambulation 4–6 h post-procedure) is feasible and safe after TF-TAVI. Additionally, early ambulation benefits the patients by decreasing the combined incidence of delirium, infections, pain and unplanned urinary catheter use, and thus it may be beneficial to adopt such a protocol into contemporary TAVI practice.

## Caption Electronic Supplementary Material

Table S1: Full checklist of exclusion criteria

## References

[1] Leon MB, Smith CR, Mack M, Miller DC, Moses JW, Svensson LG (2010). Transcatheter aortic-valve implantation for aortic stenosis in patients who cannot undergo surgery. N. Engl. J. Med..

[2] Cribier A, Eltchaninoff H, Bash A, Borenstein N, Tron C, Bauer F (2002). Percutaneous transcatheter implantation of an aortic valve prosthesis for calcific aortic stenosis: first human case description. Circulation.

[3] Leon MB, Smith CR, Mack MJ, Makkar RR, Svensson LG, Kodali SK (2016). Transcatheter or surgical aortic-valve replacement in intermediate-risk patients. N Engl J Med.

[4] Reardon MJ, Van Mieghem NM, Popma JJ, Kleiman NS, Sondergaard L, Mumtaz M (2017). Surgical or transcatheter aortic-valve replacement in intermediate-risk patients. N Engl J Med.

[5] Thourani VH, Kodali S, Makkar RR, Herrmann HC, Williams M, Babaliaros V (2016). Transcatheter aortic valve replacement versus surgical valve replacement in intermediate-risk patients: a propensity score analysis. Lancet.

[6] Popma JJ, Deeb GM, Yakubov SJ, Mumtaz M, Gada H, O’Hair D (2019). Transcatheter aortic-valve replacement with a self-expanding valve in low-risk patients. N Engl J Med.

[7] Mack MJ, Leon MB, Thourani VH, Makkar R, Kodali SK, Russo M (2019). Transcatheter aortic-valve replacement with a balloon-expandable valve in low-risk patients. N Engl J Med.

[8] Waksman R, Rogers T, Torguson R, Gordon P, Ehsan A, Wilson SR (2018). Transcatheter aortic valve replacement in low-risk patients with symptomatic severe aortic stenosis. J Am Coll Cardiol.

[9] Anand A, Harley C, Visvanathan A, Shah ASV, Cowell J, MacLullich A (2017). The relationship between preoperative frailty and outcomes following transcatheter aortic valve implantation: a systematic review and meta-analysis. Eur Heart J Qual Care Clin Outcomes.

[10] Bagienski M, Kleczynski P, Dziewierz A, Rzeszutko L, Sorysz D, Trebacz J (2017). Incidence of postoperative delirium and its impact on outcomes after transcatheter aortic valve implantation. Am J Cardiol.

[11] Soundhar A, Udesh R, Mehta A, Schindler J, Jeevanantham V, Gleason T (2017). Delirium following transcatheter aortic valve replacement: national inpatient sample analysis. J Cardiothorac Vasc Anesth.

[12] Shehada SE, Wendt D, Peters D, Mourad F, Marx P, Thielmann M (2018). Infections after transcatheter versus surgical aortic valve replacement: mid-term results of 200 consecutive patients. J Thorac Dis.

[13] Barbanti M, Gulino S, Costa G, Tamburino C (2018). Optimization and simplification of transcatheter aortic valve implantation therapy. Expert Rev Cardiovasc Ther.

[14] Wood DA, Lauck SB, Cairns JA, Humphries KH, Cook R, Welsh R (2019). The Vancouver 3M (multidisciplinary, multimodality, but minimalist) clinical pathway facilitates safe next-day discharge home at low-, medium-, and high-volume transfemoral transcatheter aortic valve replacement centers: the 3M TAVR study. JACC Cardiovasc Interv.

[15] Sawaya FJ, Lefevre T, Spaziano M, Roy A, Fernandez L, Garot P (2016). Transfemoral transcatheter aortic valve implantation: how minimalistic can we become?. J Interv Cardiol.

[16] Wiegerinck EM, Boerlage-van Dijk K, Koch KT, Yong ZY, Vis MM, Planken RN (2014). Towards minimally invasiveness: transcatheter aortic valve implantation under local analgesia exclusively. Int J Cardiol.

[17] Baan J, Vendrik J (2018). The sooner the better?: The doctor knows best. JACC Cardiovasc Interv.

[18] Kotronias RA, Teitelbaum M, Webb JG, Mylotte D, Barbanti M, Wood DA (2018). Early versus standard discharge after transcatheter aortic valve replacement: a systematic review and meta-analysis. JACC Cardiovasc Interv.

[19] Juergens C (2013). Early ambulation after percutaneous coronary intervention does not increase bleeding risk compared with late ambulation. Evid Based Nurs.

[20] Koch KT, Piek JJ, de Winter RJ, Mulder K, David GK, Lie KI (1997). Early ambulation after coronary angioplasty and stenting with six French guiding catheters and low-dose heparin. Am J Cardiol.

[21] Koch KT, Piek JJ, de Winter RJ, Mulder K, Schotborgh CE, Tijssen JG (1999). Two hour ambulation after coronary angioplasty and stenting with 6 F guiding catheters and low dose heparin. Heart.

[22] Kappetein AP, Head SJ, Genereux P, Piazza N, van Mieghem NM, Blackstone EH (2012). Updated standardized endpoint definitions for transcatheter aortic valve implantation: the Valve Academic Research Consortium-2 consensus document (VARC-2). Eur J Cardiothorac Surg.

[23] Collins SL, Moore RA, McQuay HJ (1997). The visual analogue pain intensity scale: what is moderate pain in millimetres?. Pain.

[24] Wewers ME, Lowe NK (1990). A critical review of visual analogue scales in the measurement of clinical phenomena. Res Nurs Health.

[25] Biancari F, Romppanen H, Savontaus M, Siljander A, Makikallio T, Piira OP (2018). MANTA versus ProGlide vascular closure devices in transfemoral transcatheter aortic valve implantation. Int J Cardiol.

[26] Van Mieghem NM, Latib A, van der Heyden J, van Gils L, Daemen J, Sorzano T (2017). Percutaneous plug-based arteriotomy closure device for large-bore access: a multicenter prospective study. JACC Cardiovasc Interv.

[27] Moriyama N, Lindstrom L, Laine M (2018). Propensity-matched comparison of vascular closure devices after transcatheter aortic valve replacement using MANTA versus ProGlide. EuroIntervention.

[28] Wood DA (2016). Could a “simplified” transcatheter aortic valve replacement procedure eliminate post-operative delirium?. JACC Cardiovasc Interv.

