# Supplementary material for: Early mobilisation after transfemoral transcatheter aortic valve implantation: results of the MobiTAVI trial
Source: Neth Heart J. 2020 Feb 28;28(5):240–8. doi: 10.1007/s12471-020-01374-5 (PMC7190768; doi:10.1007/s12471-020-01374-5)
Supplement: Supplementary file 1 — Table S1: Full checklist of exclusion criteria [file 12471_2020_1374_MOESM1_ESM.docx]

**Online supplemental Table S1: Full checklist of exclusion criteria**

| **Time point 1 (T1)** (preoperative, T=-1 hours) |
| --- |
| 1. pre-existent inability to walk or make independent transfers |
| 1. unable or unwilling to give informed consent |
| **Time point 2 (T2)** (per- or direct postoperative, T=0 hours) |
| 1. Any major procedural complications making early mobilization (such as inability to perform the actual TF-TAVI, unplanned general anaesthesia, major vascular complications, conversion to emergency AVR, death) |
| **Time point 3 (T3)** (T=+4 hours) |
| 1. closure device failure (or modified closure device failure), or after consultation with the operator judging the closure too difficult and early ambulation too hazardous, or vascular closure by a surgeon 2. active access site bleeding |
| 1. access site hematoma >5 cm |
| 1. suspected false aneurysm of the femoral artery (femoral, systolic murmur) |
| 1. extreme pain (VAS>8) |
| 1. symptomatic hypotension |
| (vii) rhythm problems (in consultation with the operator),  or presence of transvenous pacemaker  (viii) negative consultation with operator for other reasons  (ix) patient not willing to ambulate early |
